# Semi-supervised learning in cancer diagnostics

**DOI:** 10.3389/fonc.2022.960984

**Published:** 2022-07-14

**Authors:** Jan-Niklas Eckardt, Martin Bornhäuser, Karsten Wendt, Jan Moritz Middeke

**Affiliations:** ^1^ Department of Internal Medicine I, University Hospital Carl Gustav Carus, Dresden, Germany; ^2^ Else Kröner Fresenius Center for Digital Health, Technical University Dresden, Dresden, Germany; ^3^ German Consortium for Translational Cancer Research, Heidelberg, Germany; ^4^ National Center for Tumor Disease (NCT), Dresden, Germany; ^5^ Institute of Software and Multimedia Technology, Technical University Dresden, Dresden, Germany

**Keywords:** semi-supervised learning, cancer, diagnostics, artificial intelligence, machine learning

## Abstract

In cancer diagnostics, a considerable amount of data is acquired during routine work-up. Recently, machine learning has been used to build classifiers that are tasked with cancer detection and aid in clinical decision-making. Most of these classifiers are based on supervised learning (SL) that needs time- and cost-intensive manual labeling of samples by medical experts for model training. Semi-supervised learning (SSL), however, works with only a fraction of labeled data by including unlabeled samples for information abstraction and thus can utilize the vast discrepancy between available labeled data and overall available data in cancer diagnostics. In this review, we provide a comprehensive overview of essential functionalities and assumptions of SSL and survey key studies with regard to cancer care differentiating between image-based and non-image-based applications. We highlight current state-of-the-art models in histopathology, radiology and radiotherapy, as well as genomics. Further, we discuss potential pitfalls in SSL study design such as discrepancies in data distributions and comparison to baseline SL models, and point out future directions for SSL in oncology. We believe well-designed SSL models to strongly contribute to computer-guided diagnostics in malignant disease by overcoming current hinderances in the form of sparse labeled and abundant unlabeled data.

## Introduction

In the daily routine of cancer diagnostics, an abundance of medical data in the form of images, health records and genetic assays are gathered. Potentially, these data can serve as training input for supervised machine learning classifiers, however, the availability of large-scale labeled datasets represents a substantial bottleneck that limits the advancement of supervised learning (SL) techniques for diagnostic purposes. As the currently most popular technique in ML-guided diagnostics, SL requires data with high-quality labels to train a classifier that is subsequently tested on previously unseen data and evaluated based on its hit-rate to accurately predict labels in a test set that is withheld from training. The major obstacle in this setting is the disparity between overall available data and available data with labels. The latter is the essential prerequisite for supervised learning, however, obtaining a sufficiently large set of labeled data is time- and cost-intensive, especially in highly specialized domains as cancer diagnostics. The discrepancy between an increasing number of cancer patients in an aging society and the receding physician workforce as well as the correspondingly ever-growing workload of radiologists, pathologists and oncologists poses a further constraint on the labeling process as their experience and knowledge is needed to provide high-quality labels. Still, time and resources for the generation of such large-scale labeled data sets is often missing (1, 2). Therefore, strategies are needed that leverage the overall amount of available data while imposing manageable needs for labeling.

Conceptually, Semi-Supervised Learning (SSL) can be positioned at midway between Unsupervised Learning (UL), where no labels are provided and algorithms deconstruct patterns from unlabeled data e. g. for cluster analysis, and SL, where a classifier is trained on labeled data to correctly map labels to unseen data from the same distribution (3). Hence, SSL offers the opportunity to leverage the vast amounts of unlabeled medical data that are acquired in clinical routine to boost classification performance in a diagnostic setting without the need for fully-labeled extensive data sets. Nevertheless, there are critical assumptions for SSL to function properly and models have to be conceptualized and developed with diligence in order to actually provide a performance boost compared to SL models.

In this review, we aim to provide medical professionals with an outline of key concepts of SSL and how to apply it to medical data with a focus on oncology. First, we introduce main functionalities of SSL and delineate it from SL and UL. Subsequently, we provide an overview of SSL techniques applied to cancer diagnostics and care differentiating between image-based and non-image-based use-cases. Finally, we discuss pitfalls in SSL research design for medical applications and provide an outlook on future prospects.

## What is semi-supervised learning?

**Figure 1:**
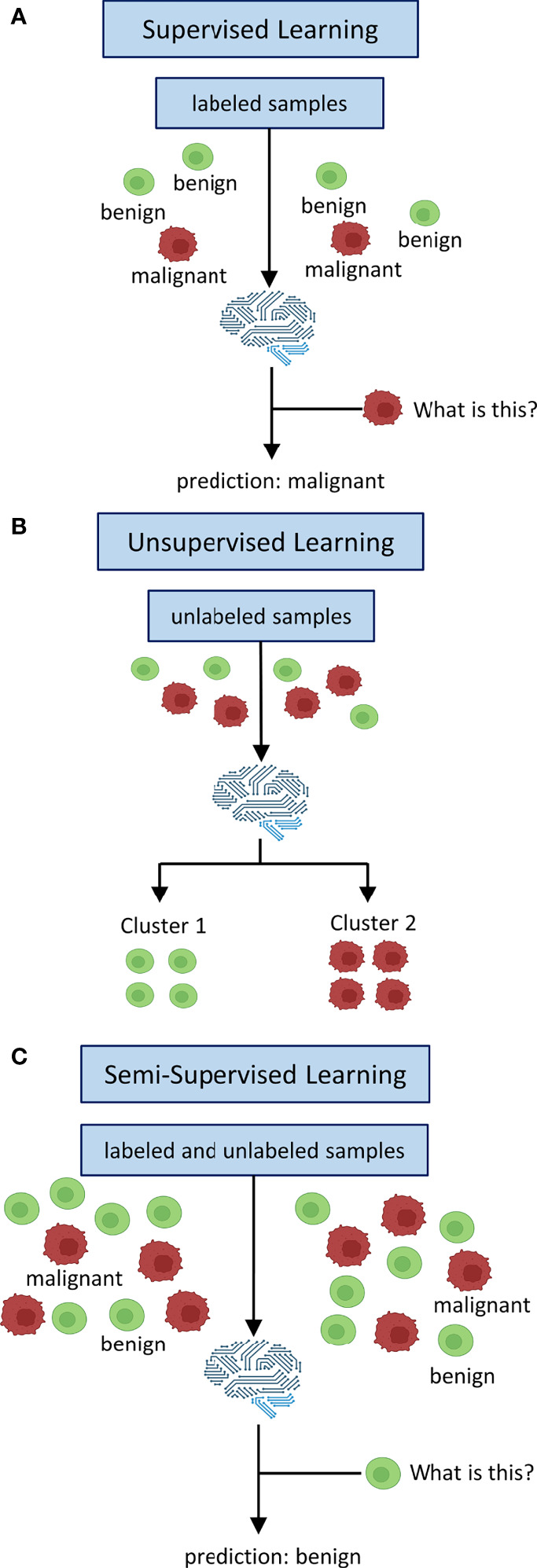
Inputs and Outputs of supervised, unsupervised and semi-supervised learning. In supervised learning **(A)** all data is labeled. Labels are used to train a classifier to map learned labels to previously unseen data. Unsupervised learning **(B)** does not use labels. Data is being clustered into groups based on inherent patterns. Semi-supervised learning **(C)** uses both labeled and unlabeled data. Labels are used to train a classifier which is augmented by unlabeled data of the same distribution to derive additional information in order to boost performance.

The key concept to delineate SL, SSL and UL is the labeling process as well as whether at all and if so, how labeled data is being processed. Labeling refers to the process of attaching meaningful information for classification to raw data. One way to do this is to have experts, e. g. medical doctors, evaluate the raw data, e. g. medical images (4). For example, whole-slide images (WSI) of tumor tissue can be labeled by pathologists or chest CAT scans for potentially malignant lesions can be labeled by radiologists. Alternatively in SSL, a limited number of labels can be used to self-train an algorithm iteratively to attach labels to unlabeled raw data and subsequently train a classifier on these self-labeled data (5). Conceptually, these labeled data provide the basis for training SL algorithms (training stage) that are subsequently supposed to apply previously learned patterns to unseen data and assign correct labels (testing stage, **Figure 1A**) (6). UL on the other hand does not use any labeled data at all. In UL, unlabeled data is sorted according to inherent patterns that delineate different clusters (7), e. g. UL can identify patient clusters with co-occurring genetic variants (**Figure 1B**). SSL uses both labeled and unlabeled data in the sense that labeled data are used to train a classifier for a given use-case and the addition of unlabeled data is intended to leverage information gain and thus boost classification performance (**Figure 1C**) (8). It is therefore advantageous when a large dataset is available for which only a limited number of labels can be obtained, i. e. due to time or cost constraints as is usually the case for medical data.

**Figure 2:**
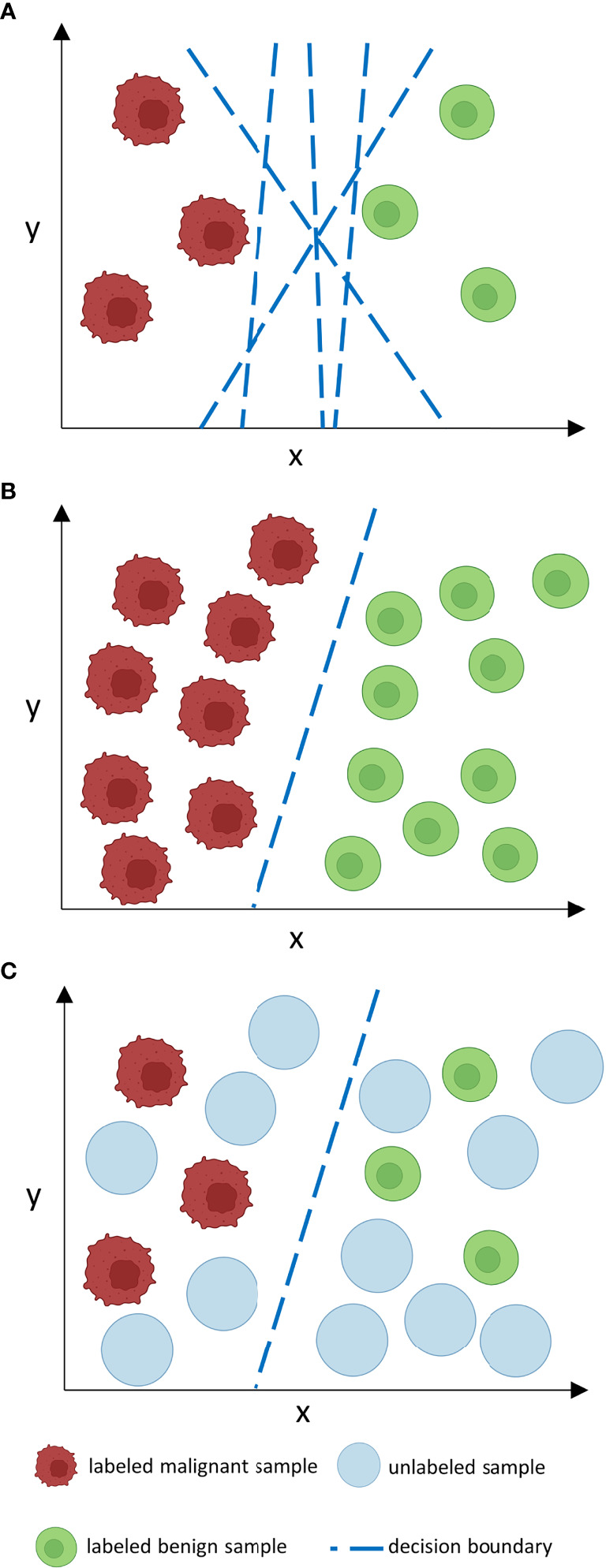
How does unlabeled data boost classification performance? Consider a number of features *n* at the input level which corresponds to an *n*-dimensional feature space. In such an n-dimensional coordinate system, every input is located according to its feature vector given by its *n* features and can thus be sorted by similarities and differences in relation to other inputs which is represented by proximity or distance points in the feature space. For clarity reasons, we only consider two features (x, y) in a two-dimensional feature space. When labeled data is sparse **(A)**, as is often the case in medical data sets, the decision boundary of a classifier is less constraint. This may lead to inaccuracies and poor generalization on external data. If many labels are given, the decision boundary is more constraint and thus a more accurate classifier is given that can potentially generalize better. However, manual labeling of such large data sets is often time- and cost-ineffective. Unlabeled data is often available in abundance **(C)** and can be used to constrain the decision boundary of a classifier in a way as large labeled data sets could do, however, without the need for excessive labeling. The decision boundary then lies in an area with low density. Nevertheless, as can be derived from **(B)** and **(C)**, the performance gap between supervised and semi-supervised learning shrinks as the amount of labeled data grows if no further unlabeled samples are provided.

While the addition of unlabeled data can be advantageous, it can also cause issues with model performance leading to stagnation or even degradation if crucial assumptions of SSL design are not met (9). For SSL models to work robustly, it is necessary that the unlabeled data should contain information that is relevant for label prediction. Therefore, it is crucial that both labeled and unlabeled data follow the same distribution (10). For example, if a classifier is trained on labeled histopathological images of colorectal cancer, the unlabeled data should ideally encompass the same tumor entity, same staining procedure and same magnification. Hence, the algorithm can infer that two samples that are close to each other at the input level (according to their features) should also be close to each other at the output level, i. e. should receive the same labels (smoothness assumption) (8). If these high-dimensional data points at the input level are mapped to a lower dimension in Euclidean space, they are usually clustered along low-dimensional structures, so-called manifolds. Data points that lie on the same manifold should therefore be of the same class (8). If both previous assumptions – inputs with similar feature vectors will be close to each other in an *n*-dimensional feature space and be located on the same manifold if mapped to a lower dimensional space - are true, the decision boundary for a classifier should then lie in an area with low density, i. e. where data points are separate and of different classes (8). Thus, the inclusion of unlabeled data (as long as it is from the same distribution as labeled data) can improve the designation of the decision boundary and therefore boost classification performance (**Figure 2**).

As is the case for most machine learning applications, there is no ‘one-size fits all’ approach and different methods and algorithms have to be evaluated for any given use-case. What further complicates model selection in SSL is a non-standardized taxonomy of methodologies which makes it harder to reproduce techniques proposed in the literature. Van Engelen et al. (3) recently proposed a taxonomy based on the distinction of inductive or transductive methods. The former encompass methods such as clustering with subsequent label assignment, pseudo-labeling or self- and co-training, i. e. methods that assign labels to unseen data and thus can potentially generalize, and the latter include graph-based methods that transfer information along connections of dataset-specific graphs only including data points in a given sample which then cannot be generalized to other data outside the specific sample (3). As for medical applications, the development of robust generalizable algorithms is desirable for utilization in clinical practice and hence most applied techniques in cancer diagnostics should be developed as inductive methods.

## Studies on semi-supervised learning in cancer diagnostics

Research efforts in applying SSL for diagnostics and care in oncology can broadly be divided data-wise by usage of images or non-image data for model development. Naturally, image-based use-cases most frequently stem from the fields of histopathology, radiology and radiotherapy, while non-image-based applications most frequently include genetic data.

### Image-based semi-supervised learning for cancer detection

#### Histopathology

In histopathology as a use-case, classification tasks using computer vision have to be divided into patch- or image-level diagnosis, i. e. whether areas with suspected malignancies should be distinguished from normal surrounding tissue or whether the sample as a whole should be labeled ‘malignant’ if any sign of neoplastic tissue is present. Importantly for model building, patch-level classification requires image segmentation *a priori* to classification, i. e. different areas of the sample have to be discriminated according to e. g. shapes, patterns and colors. Using a multi-center dataset of > 13.000 colorectal cancer WSI, Yu et al. (11) developed a mean teacher model to detect malignant patches that achieves a comparable area under the curve (AUC) compared to a multi-pathologist benchmark. They report a substantial improvement of SSL over SL when only a limited number of labels is available also validating their model on lung cancer and lymph node samples, but add that with a fully labeled set (with well above 10.000 labels) no difference between SSL and SL was detected. Similarly, Shaw et al. (12) deploy a student-teacher chain model where an iterative process of training a student model that subsequently becomes the teacher model for the following student and so on allows to utilize only 0.5% labeled data to detect colorectal adenocarcinoma from WSI. Wenger et al. (13) utilized consistency regularization and self-ensembling in order to detect and grade bladder cancer samples and report a 19% higher accuracy over baseline SL using only 3% labeled data. Jaiswal et al. (14) compared pre-trained models in detecting neoplastic infiltration of lymph node WSI and reported a high risk of overfitting after short training epochs which was tackled using ensemble learning. Addressing the challenge of variation within classes and similarities between classes, Su et al. (15) propose association cycle consistency loss and maximal conditional association to optimize the loss function reporting improved performance over learning by association on breast cancer histopathological images. Comparing SL and SSL, Al Azzam et al. (16) report similar accuracies for SSL when using only half the number of labels needed for SL in breast cancer prediction from fine needle aspirates. To grade breast cancer samples, Das et al. (17) employ a Generative Adversarial Network (GAN) where the discriminator uses an unsupervised model that is stacked over a supervised model with shared parameters to utilize both labeled and unlabeled samples. An Auxiliary Classifier GAN that divides lung cancer samples into malignant and benign patches which allows for subsequent pixel-based PD-L1 scoring is reported by Kapil et al. (18) for non-small cell lung cancer tissue needle aspirates. Both Marini et al. (19) and Li et al. (20) address the challenge of Gleason scoring prostate cancer samples. The former use a teacher-student approach with different combinations of a pseudo-labeling teacher training a student model utilizing both SSL and semi-weakly supervised learning that are compared to a student-only baseline (19). The latter use a pixel-based approach on prostate WSI with expectation maximization by a fully convolutional encoder-decoder net incorporating both internally annotated and external weakly annotated image data compared to a model trained on a fully labeled dataset alone (20). Both report performance improvements for the SSL methods using additional un- or weakly-labeled data. Lastly, to detect melanoma, Masood et al. (21) train deep belief networks in parallel to support vector machines that are supposed to counteract misclassified data with adjusted weights and finally compare their model to several SL-based models and report superior performance for their SSL-based approach. **Table 1** provides an overview of recent studies that use SSL in histopathology.

**Table 1:** Overview of Studies on Semi-Supervised Learning in Histopathology.

Authors and Reference	Entity	Objective	Technique	Publicly Available Code
Yu et al. (11)	colorectal and lung cancer as well as lymph nodes	detecting malignant patches in WSI	mean teacher	yes
Shaw et al. (12)	colorectal cancer	detecting malignant patches in WSI	student-teacher-chain	no
Wenger et al. (13)	bladder cancer	detection and grading	consistency regularization and self-ensembling	no
Jaiswal et al. (14)	metastasized tumors	detecting metastases in lymph node WSI	pseudo-labeling	no
Su et al. (15)	breast cancer	detecting malignant samples in WSI	combination of association cycle consistency loss and maximal conditional association loss	no
Das et al. (17)	breast cancer	grading samples	stacked semi-supervised GAN	no
Al Azzam et al. (16)	breast cancer	cancer detection from nuclei morphologies	comparison of 9 SL and SSL classifiers	no
Kapil et al. (18)	lung cancer	PD-L1 scoring	auxiliary classifier GAN and pixel-based quantification	no
Marini et al. (19)	prostate cancer	Gleason scoring	teacher-student chain and pseudo-labeling	yes
Li et al. (20)	prostate cancer	Gleason scoring	expectation maximization-based fully convolutional encoder-decoder network	no
Masood et al. (21)	melanoma	detecting malignant samples	Co-training of Deep Belief Network and advised SVM	no

GAN, generative adversarial networks; SL, supervised learning; SLL, semi-supervised learning; SVM, support vector machines; WSI, whole-slide-images.

#### Radiology and radiotherapy

The detection of lung nodules in computer-assisted tomography (CAT) scans is a common theme in SSL-based research in radiology. Khosravan et al. (22) use a multi-tasking CNN to concomitantly learn nodule segmentation and false positive nodule reduction on chest CAT scans incorporating SSL to accommodate for unlabeled data in the segmentation process and report high accuracies compared to baseline. Xie et al. (23) address the task of differentiating between benign and malignant nodules using a semi-supervised adversarial model with an autoencoder unsupervised reconstruction net, learnable transition layers, and a supervised classification net and report high accuracies on a benchmark dataset for lung nodule classification. Using a similarity metric function to iteratively include unlabeled samples *via* SSL, Shi et al. (24) use a transfer learning approach with a pre-trained network that differentiates between nodules and nodule-like tissue to identify lung nodules and report high accuracies in their initial dataset, but acknowledge performance drops in an independent validation set. For breast cancer detection in mammogram images, both Sun et al. (25) and Azary et al. (26) use a co-training approach. In the former study, a three-step method of adjusting weights, selecting features and co-training-based labeling is proposed and a 7.4% performance gain for the combination of labeled and unlabeled data compared to labeled data only is reported (25). The latter study incorporates SSL in pixel-based tumor segmentation and proposes co-training with support vector machines and Bayesian classifiers (26). Using breast ultrasound images for tumor detection in a joint dataset of many weakly and few strongly annotated images, Shin et al. (27) propose a self-training method and report similar accuracies for only ten strongly annotated images joined by a large number of weakly annotated ones compared to 800 strongly annotated images only. Wodzinski et al. (28) aim to identify target volumes for postoperative tumor bed irradiation in breast cancer using a semi-supervised volume penalty *via* a multi-level encoder decoder architecture and report a decrease in target registration error and tumor volume ratio. For brain tumor detection, Ge et al. (29), Chen et al. (30), and Meier et al. (31) investigate brain magnetic resonance imaging (MRI) scans. Ge et al. (29) utilize a graph-based approach to create pseudo-labels and accommodate for moderate-sized data sets by generating additional images with GANs. They use their model for glioma grading and IDH-mutation status prediction (29). In a step-wise approach, Chen et al. (30) deploy a student-teacher-based model and extract hierarchical features using an adversarial network to detect lesions in brain MRI scans that correspond to either multiple sclerosis, ischemic stroke or tumor tissue. In a pre- and postoperative comparative setting, Meier et al. (31) investigate residual tumor tissue in brain MRI scans of ten high-grade glioma patients with semi-supervised decision forest and report improved performance and computation time compared to conventional segmentation methods. Lastly, Turk et al. (32) address thyroid cancer detection in ultrasound texture data with linked clinical scoring systems as additional features using an autoencoder-based model and report a high sensitivity despite their imbalanced dataset by using synthetic minority oversampling. **Table 2** provides an overview of studies using SSL in radiology or radiotherapy.

**Table 2:** Overview of Studies on Semi-Supervised Learning in Radiology and Radiotherapy.

Authors and Reference	Entity	Objective	Technique	Publicly Available Code
Khosravan et al. (22)	lung cancer	detecting malignant nodules in chest CAT scans	SSL-based multi-task network	no
Xie et al. (23)	lung cancer	detecting malignant nodules in chest CAT scans	semi-supervised adversarial autoencoders, learnable transition layers, and supervised classification	no
Shi et al. (24)	lung cancer	detecting malignant nodules in chest CAT scans	transfer learning and semi-supervised feature matching	no
Sun et al. (25)	breast cancer	detecting breast cancer in mammogram images	co-training	no
Azary et al. (26)	breast cancer	detecting breast cancer in mammogram images	co-training	no
Shin et al. (27)	breast cancer	detecting breast cancer in ultrasound images	joint weakly- and strongly-supervised framework and self-training	yes
Wodzinski et al. (28)	breast cancer	identifying target volumes for radiotherapy	semi-supervised multilevel encoder-decoder	yes
Ge et al. (29)	brain tumor	glioma grading and IDH-mutation prediction in MRI scans	GAN-augmented networks in a graph-based framework	no
Chen et al. (30)	brain tumor, multiple sclerosis, ischemic stroke	detecting pathological samples in MRI scans	student-teacher chain combined with adversarial learning	yes
Meier et al. (31)	brain tumor	detecting residual tumor tissue in postoperative brain MRI	semi-supervised decision forest	no
Turk et al. (32)	thyroid cancer	detecting thyroid cancer from ultrasound textures and clinical scoring systems	autoencoders and synthetic minority oversampling	no

CAT, computer-assisted tomography; GAN, generative adversarial networks; MRI, magnetic resonance imaging.

### Non-image-based semi-supervised learning for cancer management

While the aforementioned image-based studies primarily focus on detection of cancer, research efforts of SSL in oncology that do not use images predominantly address the task of estimating survival, predicting relapse and identifying genetic subtypes. Examining gene expression data from patients with breast, lung, gastric and liver cancer, Chai et al. (33) use a semi-supervised self-paced learning framework with Cox proportional hazard and accelerated failure time models to classify cancer patients and predict censored data thereby reporting improved separation of survival curves for their model compared to baseline supervised models. Also using gene expression data but in the context of colorectal and breast cancer, Shi et al. (34) predict recurrence *via* low density separation. They report increasing accuracies for SSL over baseline SL classifiers with increasing numbers of unlabeled data. Addressing the same task in the same tumor entities, Park et al. (35) resort to a semi-supervised graph regularization algorithm to identify functionally similar gene pairs and thereby predict recurrence in breast and colorectal cancer gene expression data including labeled and unlabeled nodes. Hassanzadeh et al. (36) designed an ensemble model based on decision trees and boosting to predict survival for patients harboring kidney, ovarian, or pancreatic cancer for whom only incomplete clinical data was available and report improved accuracy for SSL compared to SL baselines. Cristovao et al. (37) compared SL and SSL in subtyping breast cancer using multi-omic data, however, did not find any performance improvements when comparing SSL to baseline logistic regression. Also investigating multi-omics data, Ma et al. (38) developed affinity fusion networks to cluster patients based on their specific omics profile into lung, kidney, uterus or adrenal gland cancer groups. The authors report a high predictive accuracy with training on less than one percent of labeled data. Sherafat et al. (39) developed a positive-unlabeled learning model using auto machine learning to predict tumor-rejection mediation neoepitopes from exome sequencing data in ovarian cancer. The authors report improved performance over model-based classifiers for somatic variant calling and peptide identification. Both Camargo et al. (40) and Livieris et al. (41) propose novel active learning models that are tested on either data of acute myeloid leukemia, E. coli, and plant leaves, or breast and lung cancer, respectively. In both studies, the authors report higher accuracies for their respective models, root distance boundary sampling (40) and improved CST voting (41), compared to both SSL and SL classifiers. **Table 3** summarizes non-image-based applications of SSL with relevance to cancer detection and management.

**Table 3:** Overview of Studies on Semi-Supervised Learning using non-image-based data.

Authors and Reference	Entity	Objective	Technique	Publicly Available Code
Chai et al. (33)	breast, lung, gastric and liver cancer	predicting survival	self-paced learning with Cox proportional hazard and accelerated failure time models	no
Shi et al. (34)	colorectal and breast cancer	predicting relapse	low density separation	no
Park et al. (35)	colorectal and breast cancer	predicting relapse	graph-based regularization	no
Hassanzadeh et al. (36)	kidney, ovarian and pancreatic cancer	predicting survival	ensemble learning with robust boost and decision trees	no
Cristovao et al. (37)	breast cancer	subtyping, model comparison	comparison of different SL and SSL algorithms	no
Ma et al. (38)	lung, kidney, uterus and adrenal gland cancer	predicting primary tumor site	Affinity Network Fusion	yes
Sherafat et al. (39)	ovarian cancer	predicting tumor-rejection mediating neoepitopes	Positive-unlabeled Learning using Auto-ML	no
Camargo et al. (40)	acute myeloid leukemia, E. coli, plant leaves	model comparison	root distance boundary sampling	yes
Livieris et al. (41)	breast and lung cancer	model comparison	self- and co-training with ensemble learning	no

## Discussion

SSL represents a viable approach to the dilemma of big data in cancer medicine, especially in the context of image data which is usually acquired in abundance during clinical routine work-ups, but adequate labeling by medical experts is often time consuming and thus cost-ineffective. The main goal of SSL in this context is to achieve classification performances that surpass those of SL alone when labeled data is limited and at the same time abundant unlabeled data is available. Crucially, SSL models have to satisfy the above-mentioned assumptions: i) both labeled and unlabeled data have to be drawn from the same distribution, ii) similarity of data on the input level results in similarity of data at the output level (smoothness), iii) hence data points on the same low-dimensional structures (manifolds) receive the same labels and thus, iv) the decision boundary runs through an area of low density, i.e. where data points are separated and of different classes. Divergence from these key assumptions can not only lead to performance stagnation, but also degradation as unlabeled data is handled as noise that blurs information abstraction of the classifier (42). Importantly, this is what delineates SSL from transfer learning, where a classifier is first trained on one use-case and subsequently transferred to another similar use-case where it is supposed to perform a similar task (43), e. g. a classifier trained by identifying alteration A in immunohistochemistry on WSI in a supervised setting could potentially be transferred to also identify alteration B if staining is similar. Therefore, the most important question before conducting SSL experiments is whether labeled and unlabeled data are actually from the same distribution and if so whether an inclusion of the unlabeled samples might lead to a performance gain over baseline SL.

Several of the above-mentioned studies reported substantial performance gains for SSL as long as the model was short on labeled data, however, when the amount of labeled data was increased or only labeled data was used the gap between SSL and SL performance shrunk. However, the frequent lack of a comparison between baseline SL and SSL classifiers further complicates the evaluation of such studies and only few studies do report baseline comparisons (11, 13, 19, 22, 33, 37) and still even fewer report equal tuning of hyperparameters (11, 19) for SSL and SL classifiers to make results comparable. When it comes to model design, it is essential to note that different algorithms may perform differently with regard to different tasks (9). While this sounds obvious, it is still the case that often only the use of a single algorithm is reported which either may be due to a lack of comparative testing or due to publication bias as only the successful algorithm is selected for a given manuscript. However, to evaluate suitable model designs for different tasks, we advocate for a full report on tested algorithms ideally including a comparison between different SSL model set-ups, their SL baseline, adequate hyperparameter tuning for both SSL and SL, and the models’ individual performance in comparison. Further, varying the amount of labeled and unlabeled data for both training and testing sets seems warranted to find the equilibrium of optimal performance for different tasks in future studies of SSL in oncology. The lack of reproducibility in research on artificial intelligence in general (44) is also likely to be a future issue in biomedical use-cases of SSL as unfortunately only a minority of studies provide publicly accessible code to support their results (11, 19, 27, 28, 30, 38, 40). As is evident from previous studies on SSL in oncology, use cases mainly include tumor entities with high prevalence such as breast (15–17, 25–28, 33–35, 37, 41), lung (18, 22, 23, 33, 34, 38, 41), and colorectal cancer (11, 12, 34, 35) where single centers can amass sufficiently sized data sets to conduct SSL experiments. This is also reflected in the overwhelming absence of studies on SSL in hematology with only one single study (40) including any hematological neoplasm at all. Therefore, data-sharing is crucial in order to expand use-cases to rare tumor entities. Slight differences between centers in how training data is handled – e.g. differences in imaging devices used and thus consecutive differences in image format, shape, contrast, resolution and brightness – may also influence individual models. A model trained solely on single center image data may therefore significantly drop in performance if it is introduced to data of another source. Hence, pooling heterogenous data of different sources for initial model training is useful in order to obtain classifiers that can be widely generalized beyond in-house use for single institutions. Not only may the crowd-sourcing of research in biomedical SSL vastly enlarge the pool of unlabeled (and possibly labeled) data, but it may also help identify and modify promising models for multi-center prospective validation. The latter is another key shortcoming of previous studies that were often confined to single centers and retrospective evaluation. Thus, publicly available code, data-sharing for both labeled and unlabeled data and prospective collaborative research efforts will be key to evaluate models for future clinical applicability. Shared data and models may then also enable the evaluation of a variety of tumor entities in the same diagnostic modality, i. e. differential diagnosis of tumor entities in histopathological WSI.

This, however, leads to a frequent problem of artificial intelligence in general that is even more pronounced in the sensitive context of oncology where diagnostic accuracy is essential to provide high quality care to patients with life-threatening diseases: explainability of ML models. ML and especially deep learning has often been referred to as a ‘black box’ (45) and the path of decision making within a model is hard to interpret. While this is already a key issue in SL, SSL adds to the confusion as information is also derived from unlabeled samples. The apparent lack of interpretability when it comes to clinical validation of model outputs stresses the urgent need to incorporate mechanisms of explainability into SSL models that make outputs or even intermediate steps such as label assignment on unlabeled samples traceable for clinical experts. The virtual lack thereof in previous studies signals a discrepancy between what is technologically possible and what is clinically acceptable for routine use as ‘black box’ models will likely have it harder to be included in routine clinical workflows due to a lack of acceptance in diagnostic specialties and ethical concerns in cancer management (46). Still, given large unlabeled data sets that often are routinely acquired in cancer diagnostics combined with the trend of a shrinking physician workforce that is occupied with complex tasks that have to be performed in increasingly shorter periods of time (1), SSL provides a low-cost and potentially high-benefit solution to develop clinically meaningful ML models for diagnostic tasks in oncology.

## Conclusion

While SSL provides a possible solution to the vast discrepancy between available labeled and unlabeled data in cancer diagnostics, it should not be considered a silver bullet in the development of accurate classifiers for cancer detection. Adequate selection of labeled and unlabeled data of the same distribution as well as comparisons to baseline SL, among others, are crucial to build robust SSL models. While previous research efforts of SSL in oncology have mainly comprised retrospective single-center studies, future research is warranted in multi-center prospective model evaluation to design robust and explainable classifiers for implementation in the clinical routine of cancer diagnostics.

## Author contributions

J-NE performed the literature search and wrote the initial draft. All authors provided critical scientific insights, reviewed and edited the draft and approved its final version for submission. All authors agree to be accountable on the contents of the work. All authors contributed to the article and approved the submitted version.

## Funding

J-NE is grateful for a research scholarship from the Mildred-Scheel-Nachwuchszentrum Dresden (German Cancer Aid). The funder had no role in the design and conduct of the study, analysis, and interpretation of the data, preparation, review, or approval of the manuscript; and decision to submit the manuscript for publication.

## Conflict of interest

The authors declare that the research was conducted in the absence of any commercial or financial relationships that could be construed as a potential conflict of interest.

## Publisher’s note

All claims expressed in this article are solely those of the authors and do not necessarily represent those of their affiliated organizations, or those of the publisher, the editors and the reviewers. Any product that may be evaluated in this article, or claim that may be made by its manufacturer, is not guaranteed or endorsed by the publisher.
